# The efficacy of an online self‐administered single session intervention to promote growth mindset in adolescents: A randomised controlled trial

**DOI:** 10.1002/jcv2.70026

**Published:** 2025-07-08

**Authors:** Jessica Ball, Richard Meiser‐Stedman, Maria Loades, Amorette Perkins, Gemma Bowers, Laura Pass, Joseph Cassidy, Kenny Chiu

**Affiliations:** ^1^ Department of Clinical Psychology and Psychological Therapies Norwich Medical School University of East Anglia Norwich Research Park Norwich UK; ^2^ Department of Psychology University of Bath Bath UK; ^3^ Norfolk and Suffolk NHS Foundation Trust Norwich UK

**Keywords:** growth mindset, randomised controlled trial, self‐administered, single‐session interventions, youth

## Abstract

**Background:**

Single‐session interventions (SSIs) are emerging as one promising way to support one's mental health. Growth mindset refers to the beliefs about the malleability of traits and attributes. Building upon a feasibility study of a growth mindset single session intervention, this randomised controlled trial aimed to evaluate its efficacy when delivered online to young people.

**Methods:**

We recruited participants aged 14–18‐year‐olds via social media, schools, and charities in the UK. They were randomised to receive either an online video‐based intervention or were placed on a waitlist control. They reported anxiety and depression symptoms, as well as personality mindset and psychological flexibility at baseline and at 1‐month follow up. An intention‐to‐treat (ITT) analysis and a case completer analysis were conducted.

**Results:**

In a sample of 104 participants (mean age = 16.3), an ITT analysis yielded negligible effects on anxiety and depression symptoms (*d* = 0.07, 95% CI: [−0.32, 0.47]) and psychological flexibility (*d* = −0.12, 95% CI: [−0.50, 0.25]) at 4‐week follow‐up. The personality mindset measure yielded a significant large effect relative to waitlist (*d* = −0.96, 95% CI: [−1.87, −0.04], *p* = 0.02), however it was statistically non‐significant with Bonferroni correction. Case completer analysis resulted in similar observations.

**Conclusion:**

The intervention impacted personality mindset but had limited effect on anxiety and depression. Large sample sizes, improve retention rate, and a longer follow‐up period are needed in future studies.

## Graphical abstract

We examined the efficacy of an online growth mindset single session intervention (SSI) for young people aged 14–18 years old in the UK. 104 participants were randomised to a registered clinical trial. Intention‐to‐treat analysis yielded negligible effect sizes for anxiety and depression symptoms, whereas the effect on personality mindset was statistically significant.

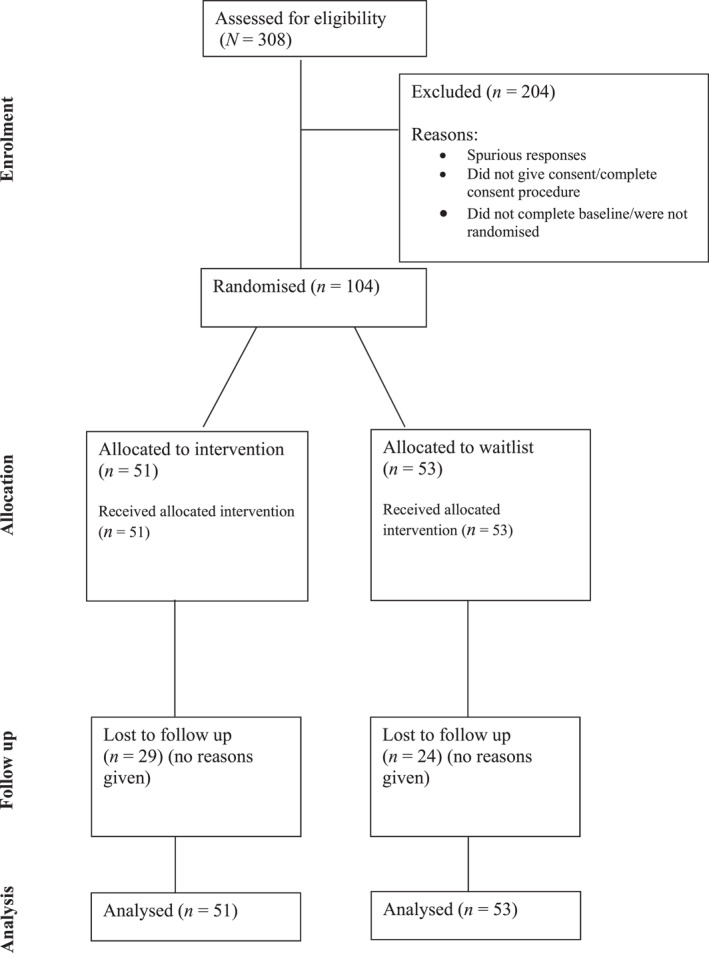

**Key points**

- Online Single Session Interventions (SSIs) are a promising way to improve young people's access to mental health support.
- This randomised controlled trial (RCT) evaluated an online SSI for youth aged 14–18 years.
- The intention‐to‐treat analysis showed no significant effects on anxiety and depression.
- It yielded a significant large effect on personality mindset.
- Low retention, small sample size, short follow‐up period limited conclusions.
- The study highlights the potential scalability of online SSIs.

## INTRODUCTION

Adolescent mental health is an area of great concern worldwide, with up to 20% of young people living with a clinically significant mental health condition (World Health Organisation [WHO], 2003; Public Health England & Children and Young People's Mental Health Coalition, 2015). In the UK, one in five young people are reported to have a probable mental health disorder (NHS Digital, 2023), yet 75% of those who need support are not receiving it (National Health Service [NHS] Digital, 2021). Most mental health conditions in adult life develop during childhood or adolescence (Kessler et al., 2005). It is important to promote positive emotional wellbeing and prevent conditions from developing in the first instance (Department of Health and Social Care, 2015; Public Health England, 2019).

Single‐session interventions (SSIs) are described as a single encounter with an intervention that is intended to achieve treatment dose in one session. Unlike conventional digital interventions, which often experience dropouts over time (Loades & Schleider, 2023), SSIs can be delivered in various ways, including by parents or schools, or by mental health professionals through various platforms such as clinics, phones or even chatbots (Schleider & Weisz, 2017). They could be one promising way to expand access to evidence‐based interventions (Schleider et al., 2020). A meta‐analysis by Schleider and Weisz (2017) highlighted SSIs as having the largest effect sizes for anxiety and conduct problems and these effects did not differ between self‐ versus therapist‐administered SSIs. They also found that young people who received an SSI were 58% more likely to have better outcomes than participants who received no intervention. Online, self‐guided SSIs may be uniquely scalable if they are freely available for use as needed (Schleider et al., 2020).

SSIs can be informed by a range of underlying theoretical approaches to support mental health, one being through promotion of an adaptive mindset. Mindset is defined as ‘the fundamental, core beliefs that individuals hold about the nature and malleability of various aspects of the human condition’ (Ryan & Mercer, 2012, p. 74). Growth mindset, in particular, is underpinned by theories of motivation, focusing on how individuals respond to setbacks and challenges, with the view that we have the potential to change, relative to our prior abilities (Yeager & Dweck, 2020). Growth mindset has been explored within educational settings, but is increasingly gaining attention in mental health research (Burnette et al., 2020).

A systematic review by Burnette et al. (2022) suggests that the effects of mindset interventions on mental health are stronger than the effects for academic achievement, but the number of studies on mental health outcomes is limited. Having a growth mindset matters as research suggests those who hold a growth mindset are more likely to prosper in the face of difficulty compared to those who have a fixed mindset, who may be wary of challenges or fail to meet their potential due to fear of failure (Dweck & Yeager, 2019). Furthermore, there is evidence of a positive link between growth mindsets and coping across clinical and non‐clinical populations (Burnette et al., 2020). Having a fixed mindset is linked to experiencing more mental health problems (Schroder et al., 2019) and increased psychological distress (Burnette et al., 2020). Furthermore, fixed mindsets have been associated with poorer outcomes such as young people performing worse academically (Yeager & Dweck, 2020).

Following the Coronavirus pandemic, much of the world has moved to an online model of working, making it a widely adopted way to reach young people *en masse* (Hawke et al., 2021). Digital media is also a space where the young generations feel more confident and equipped to engage with mental health promotion (Prescott et al., 2019). Furthermore, digital guided self‐help has now been recognised by the National Institute of Clinical Excellence (NICE) Early Value Assessment as a recommended tool for young people (NICE, 2023).

Only two digital growth mindset SSI feasibility studies have been conducted in the UK (Ching et al., 2022; Perkins et al., 2021), but neither of them adopted a randomised controlled trial (RCT) design. In their feasibility study, Ching et al. (2022) explored the impact of a digital growth mindset SSI, based on a similar intervention to Perkins et al. (2021) and found small effects for anxiety and depression outcomes measured at 1‐month follow‐up. However, they were limited by their small sample which constituted only paediatric patients on a mental health waitlist and had no control group. A study by Perkins et al. (2021) based on a video promoting a growth mindset found that such an intervention can be feasible, acceptable, and potentially scalable. They recruited 16–18‐year‐olds and administered the 30‐min SSI via the Internet within a classroom setting, whilst those randomised to the waitlist condition continued with their normal timetabled school activities. However, limitations included lengthy questionnaires, and the sample was restricted by the schools who chose to take part, potentially causing an underrepresentation such as across ethnic groups. Also, being delivered in a school context produced burden for schools which could be reduced by moving to an online delivery model. Finally, the option for young people to opt for earlier access to interventions, than the age in Perkins et al. (2021), could be beneficial as mindsets develop and potentially become ingrained over time (Limeri et al., 2020).

The current study aimed to build on the previous feasibility study (Perkins et al., 2021). To the authors' knowledge this is the first pre‐registered RCT of an online SSI in the UK. It evaluated the efficacy of a low cost, solely online, self‐help single‐session growth mindset video intervention. It explored whether outcomes are indicative of positive change in anxiety and depression symptoms, beliefs about growth in mindset and psychological flexibility.

## METHOD

### Design

The present study is a RCT, with parallel groups and an intended allocation ratio of 1:1. The intervention group watched the intervention SSI immediately, whereas a waitlist control group accessed the intervention at the end of the study.

### Ethical considerations

Ethical approval was granted by the University of East Anglia Faculty of Medicine and Health Sciences Research Ethics Committee (ETH2223‐0067). A key ethical consideration was obtaining consent from participants aged 14–18 only. We considered the principle of Gillick competence, which has been used in various contexts, including COVID‐19 vaccination (UK Health Security Agency, 2021) and medical procedures (Shaw, 2001) in the UK context. Our decision to proceed without parental consent was justified by the non‐invasive nature of the intervention, the importance of respecting adolescents' growing autonomy in mental health decision‐making, and the potential for parental consent to introduce selection bias. The prior feasibility trial (Perkins et al., 2021) demonstrated acceptability and safety, with no adverse events reported, further supporting the appropriateness of the consent procedures.

### Participants

**FIGURE 1:**
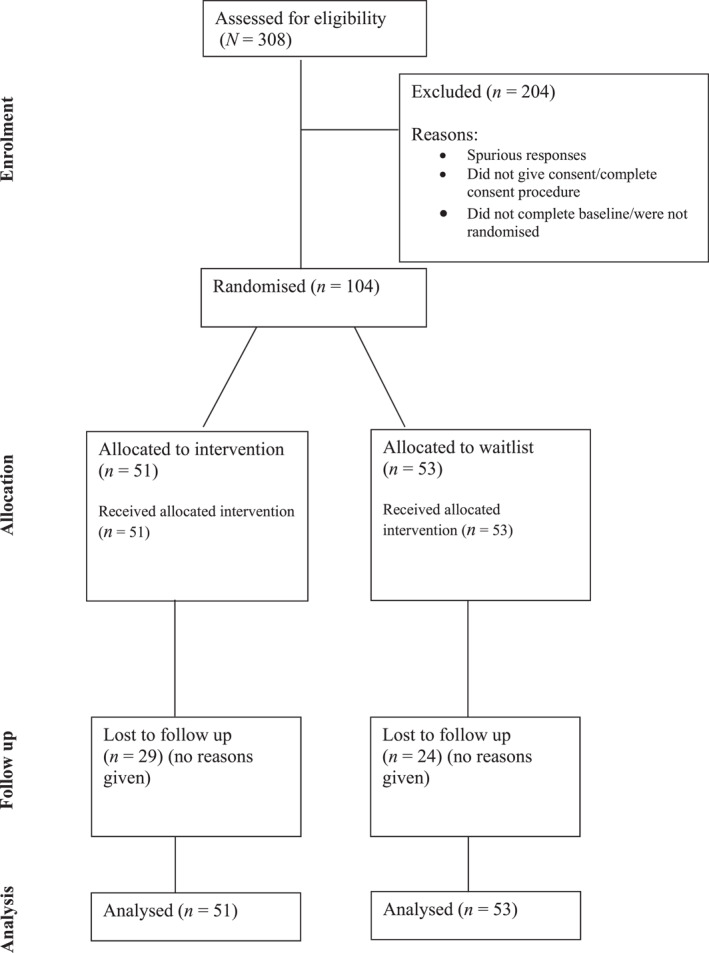
CONSORT study flow diagram.

Participants were deemed eligible if they were aged 14–18 years old, were based in the UK, and could read and write in English. They were offered the chance to enter a prize draw to win 1 of 15 shopping vouchers (worth £10 each) following completion of their follow‐up questionnaires. They were initially recruited through online social media platforms (Facebook and Instagram). Paid targeted adverts costing £158 in total, ran from the 1^st^ February 2023 to 30th April 2023 at specific times of day, for example, after school so that the study advertisement was displayed to the correct age groups at peak times on their social media, and during half terms more frequently (see Table S1). Study information was also shared online through social media accounts. The initial recruitment effort resulted in 538 young people who progressed from the advertisement to the project information page (see Figure S1). One participant was removed from the intervention arm after randomisation due to being unable to match them to baseline data. Due to a low uptake, recruitment was extended to schools and charities. Initially local mainstream schools and colleges were contacted, before reaching out to schools nationally. A total of 473 schools were contacted via email and telephone correspondence, two schools agreed to meet and discuss the project and share, and approximately a further six shared the project. Eight local third sector organisations and charities were also contacted, and one agreed to share project details. A power analysis indicated a minimum sample size of 128 was required to detect a medium effect size 0.25 with 80% power. A total of 308 participants were recruited between February 2023 and January 2024, yet 66.2% of them did not give full consent. The final sample consisted of 104 participants (see CONSORT diagram in Figure 1).

### Procedure

Interested participants clicked a link for study information provided on Qualtrics. They read the information sheet and consent form, then ticked to consent to take part. Once consented, they completed a questionnaire and were randomised either to the treatment arm or waitlist control (WLC) arm using a single‐blind approach. Those who were allocated to the treatment arm watched a ten‐minute psychoeducation video on their own device, followed by 5 minutes of stories from fictional students about how they apply the concepts from the animation.

The intervention integrated concepts from Cognitive Behavioural Therapy, Compassion Focused Therapy and Acceptance and Commitment Therapy to increase malleability of mindsets by targeting unhelpful beliefs/consequences (e.g. perfectionistic striving, self‐blame or decrease in acceptance [Dweck et al., 1995; Hoyt & Burnette, 2020; Kneeland et al., 2016; Tamir et al., 2007]). The intervention encourages one to take a compassionate stance, recognising that one cannot always stay in control of momentary psychological experiences (see Table 1 for further information about the intervention). Participants then completed three multiple choice questions aimed to assess their understanding and ability to apply the learnt concepts, followed by a optional ‘letter of advice’ task where participants wrote advice to a fictional student struggling with their psychological experiences. All participants were contacted by email 4 weeks post‐randomisation for a follow‐up assessment where they were asked to complete measures again and the WLC group could then access the intervention. The first part of the intervention took on average 17 min to complete, and the follow up took 6.5 min.

**TABLE 1:** Detailed description of intervention.

Underlying concept	Overview of content
Information on brain activity and neuroplasticity based on neuroscience	Thoughts, emotions, and behavioural urges are underpinned by complex neurological processes. We do not have complete control of in‐the‐moment experiences due to the rapid and complex nature of neuronal activity. There is also an enormous number of pathways in the brain, and we can have thousands of different experiences in our mind every day, and these are not fixed, instead transient. Our brain also has the ability to change its structure and functions in response to experiences (neuroplasticity)
CBT principles	We make sense of the world based on our previous experiences. Our thoughts, emotions, behaviours, and physiological responses are all connected and can influence one another. We can get stuck in repeated patterns of behaviour because at one time it was helpful for our survival/to protect us. At times we can continue to behave in ways that are either helpful or unhelpful
CFT and ACT principles	Humans are not perfect, and everyone has different strengths and limitations. We can learn to be compassionate towards ourselves when we experience difficulties. We can choose to not get fused with our thoughts and feelings that can bother us, instead we have the ability to change our relationship with them
	Changing our behaviour to live in accordance with our values can enhance our wellbeing
Growth mindset	Growth mindset is underpinned by theories of motivation, in particular how we respond to setbacks and challenges, with the view that we have the potential to change, relative to our prior abilities (Yeager & Dweck, 2020). Change is possible as emotions (along with other concepts such as personality and intelligence) are not fixed states, instead they are transient and malleable

Abbreviations: ACT, acceptance and commitment therapy; CBT, cognitive behavioural therapy; CFT, compassion focused therapy.

### Measures

All measures including demographic details were taken at baseline before randomisation, then again (except demographics) at the 4‐week follow‐up.

#### Demographic characteristics

Participants' age, gender identity, ethnicity, and location were collected. A question ‘do your parents own their own home?’ was asked to assess participant's socioeconomic status.

#### Treatment adherence

Qualtrics tracked the length of time spent on the webpage and did not allow participants in the intervention arm to proceed until the video finishes. Therefore, we can be certain that those participants who were randomised to intervention and completed their follow‐up watched the video.

#### Primary outcome: Anxiety and depression symptoms

The 11‐item Revised Child Anxiety and Depression Scale (RCADS‐11; Radez et al., 2021) was used to measure anxiety and depression symptoms. It contains six items on anxiety symptoms (e.g. ‘I worry when I go to bed at night’) and five on depression symptoms (e.g. ‘I have no energy for things’), using a Likert scale from 0 (Never) to 3 (Always) with a maximum score of 33. Higher scores indicate higher reported symptoms with cut off scores provided, differentiated by gender. The RCADS‐11 developers proposed the following cut off scores: Anxiety symptoms (male ≥5, female ≥9), depression symptoms (male ≥8, female ≥9), total anxiety/depression scale symptoms (male ≥9, female ≥14).

The 11‐item RCADS attained sensitivity/specificity values >0.75 (Radez et al., 2021), which are comparable to the full 47‐item RCADS (Chorpita et al., 2000) and it can be used to differentiate between community and clinic referred samples accurately. In the present study the Cronbach's alpha of this scale was *α* = 0.91.

#### Secondary outcome: Personality mindset

As per the feasibility study, three items from the Implicit Personality Theory Questionnaire (IPTQ) were used to assess respondent's view on whether their personality is fixed or malleable (Yeager et al., 2013). The self‐report three items are: ‘You have a certain personality, and it is something that you can't do much about’, ‘Your personality is something about you that you can't change very much’, and ‘Either you have a good personality, or you don't, and there is really very little you can do about it’. Items are rated on a Likert scale from 1 (really disagree) to 6 (really agree), with a higher score suggesting a more fixed mindset. Cronbach's alpha of this scale was *α* = 0.80.

#### Secondary outcome: Psychological flexibility

The Acceptance and Fusion Questionnaire for Youth‐Short Form (AFQ‐Y8; Greco et al., 2008) was used to capture third‐wave cognitive behavioural constructs assessing psychological flexibility (being present, aware, and accepting of our thoughts and emotions) and acting on values rather than short‐term impulses (Hayes et al., 2011; Hülsheger et al., 2013; Neff, 2003).

The AFQ‐Y8 is an eight item self‐report measure which uses a Likert scale from 0 (not at all true) to 4 (very true). Some example items are: ‘My life won't be good until I feel happy’ or ‘I am afraid of my feelings’. Possible scores range from 0 to 32. There are no clinical cut off scores. Lower total scores indicate greater psychological flexibility. It is validated for use within the adolescent population with reported reliability being 0.83 (Greco et al., 2008), and in this trial *α* = 0.87.

### Data analysis

Demographic data were analysed descriptively. Missing data was handled by multiple imputation following procedures outlined by Harrer et al. (2023). For the primary analysis, an Intention‐to‐treat (ITT) analysis was conducted in R Studio (R Studio Team, 2024). For the primary analysis, a series of Analysis of Covariance (ANCOVA) were conducted to explore differences between post‐treatment outcome measures for participants in the intervention and waitlist control arm, whilst controlling for baseline outcome scores and Effect sizes (Cohen's *d*) were calculated. Finally, the standardised mean difference estimated by the coefficient of the group was divided by the pooled standard deviation (Harrer et al., 2023). A sensitivity analysis was conducted using Case Completer data. Effect sizes are reported as Partial Eta Squared (*n*_*p*_^2^). Bonferroni correction was applied to account for multiple comparisons as it is a widely used and conservative method (Armstrong, 2014). To explore individual responses to the intervention, Reliable Change Index (RCI) was calculated for all outcome measure scores pre‐post treatment change for the case completer sample (Jacobson & Truax, 1991).

### Missing data management

There was no missing baseline data, however 51%–55.8% of follow up data were missing across measures, with the IPTQ missing the most responses and RCADS the least (see Table 4 for breakdown). Little's (1988) test of Missing Completely At Random (MCAR) test was non‐significant (*X* ^*2*^ = 9.249, *df* = 9, *p* = 0.411) suggesting the null hypothesis that data were MCAR cannot be rejected. For ITT analysis, Multivariate Imputation by Chained Equations (MICE) was used to impute missing data, with 50 iterations. Although over 50% of follow up data was missing, multiple imputation was employed as it has been shown to reduce bias, compared to only examining case completer data (Woods et al., 2023). Following multiple imputation, assumptions for a between‐subjects ANCOVA were tested prior to analyses. Variances were homogenous as tested by Levene's test of Equality Variances, and normality was confirmed using Shapiro‐Wilk test. There was homogeneity of regression slopes, and across all imputed data and the original data set, results were non‐significant, suggesting assumptions for ANCOVA were met.

## RESULTS

### Demographic characteristics and outcome measures at baseline

The descriptive characteristics of the 104 participants are reported in Table 2. Participants had a mean age of 16.3 (range: 14–18), and most reported being White (82.9%). Seventy‐five percent of participants reported that their parents owned their own home. In terms of how participants heard about the study, 47.1% reported via school, 34.6% did not report this information, 7.7% for ‘other’, 4.8% Facebook, 3.8% Twitter, and 1.9% Instagram.

**TABLE 2:** Baseline participant characteristics.

Participant characteristics	All participants *N* = 104	Treatment *N* = 51	Waitlist *N* = 53
Age, *M* (SD)	16.3 (1.38)	16.18 (1.45)	16.38 (1.30)
Gender, *n* (%)			
Female	61 (58.7%)	29	32
Male	22 (21.2%)	12	10
Non‐binary	12 (11.5%)	4	8
Prefer not to say	4 (3.8%)	3	1
Transgender	5 (4.8%)	3	2
Ethnicity, *n* (%)			
Asian or Asian British	7 (6.7%)	0	7
Black, Black British, Caribbean, or African	3 (2.9%)	2	1
Mixed or multiple ethnic groups	6 (5.8%)	3	3
Other ethnic group	2 (1.9%)	0	2
White	86 (82.7%)	46	40
Parents own their own home, *n* (%)			
Yes	78 (75%)	40	38
No	26 (25%)	11	15

Participant flow is summarised in the CONSORT diagram (Figure 1), with 51 participants randomised to the immediate treatment arm (22 completed follow up), and 53 to the waitlist arm (29 completed follow up). Of those randomised to the treatment arm 48% scored above recommend cut‐off for anxiety, 19% for depression, and in 55.8% of participants total anxiety/depression score was above clinical threshold. In terms of the waitlist arm, 56.6% scored above cut off for anxiety, 37.7% for depression, and 67.9% for total RCADS score. No significant group differences were observed across demographics and baseline outcome measures; therefore, randomisation was considered successful. There were also no significant group differences for those who were retained at follow‐up compared to those lost to follow‐up.

### Treatment adherence

There were 23 out of 51 (45%) participants in the intervention arm who also completed the optional letter to a student task and follow‐up questions. Those who completed the letter writing task often mentioned the brain and how it can adapt, and how emotions are transient, and wrote on average 183 words (SD = 122.5, range = 53–460).

### Intention‐to‐treat analysis

The pooled results for both primary and secondary outcomes are displayed in Table 3 the results were all non‐significant with negligible effect sizes, except for IPTQ which demonstrated a significant, large effect.

**TABLE 3:** Intention to treat analysis ANCOVA of primary and secondary outcomes.

Outcomes	*F*	*df*	*p*	*d*	95% CI
Primary outcome					
RCADS total	0.07	1, 28.66	0.794	0.07	−0.32, 0.47
Secondary outcomes					
RCADS anxiety total	0.985	1, 11.52	0.341	−0.13	−0.60, 0.34
RCADS depression total	0.027	1, 5835.68	0.869	0.14	−0.12, 0.40
AFQ total	2.739	1, 15.54	0.118	−0.12	−0.50, 0.25
IPTQ total	8.706	1, 7.29	0.020	−0.96	−1.87, −0.04

Abbreviations: 95% CI, 95% confidence interval; *d*, Cohen's *d*; df, degrees of freedom; *F*, *F* statistic; *p,* significance value.

### Case completer analysis

Differences between groups outcome measure scores for Time 1 and Time 2 for all case completers are presented in Table 4.

**TABLE 4:** Descriptive statistics for case completers.

Outcomes	Group	Pre‐Tx mean (SD)	Pre‐Tx N	Post‐Tx mean (SD)	Post‐Tx N	*p*
RCADS total	Intervention	17.6 (8.2)	51	17.6 (7.2)	22	0.766
	Waitlist	18.7 (7.1)	53	19.9 (7.3)	29	
RCADS anxiety total	Intervention	9.7 (4.5)	51	8.9 (4.3)	22	0.450
	Waitlist	10.3 (4.1)	53	10.6 (3.9)	29	
RCADS depression total	Intervention	7.8 (4.5)	51	8.7 (3.6)	22	0.337
	Waitlist	8.5 (3.6)	53	9.3 (3.9)	29	
AFQ total	Intervention	14.5 (7.9)	51	13.9 (7.4)	19	0.850
	Waitlist	16.9 (7.7)	53	17.9 (6.9)	28	
IPTQ total	Intervention	9.5 (3.6)	51	7.3 (3.2)	18	0.091
	Waitlist	10.9 (3.7)	53	11.3 (3.7)	28	

Abbreviations: *M*, mean; *N*, number of participants; SD, standard deviation.

### Primary outcome

#### RCADS total

The ANCOVA indicated a statistically non‐significant effect on RCADS scores, *F* (1, 48) = 0.090, *p* = 0.766, *n*_*p*_^2^ = 0.002. Inspection of adjusted means for RCADS scores showed that the intervention group (*M* = 19.12, SE = 0.85), did not exhibit a statistically significant difference in post intervention scores compared to the WLC (*M* = 18.78, SE = 0.74).

### Secondary outcomes

#### RCADS anxiety total

The ANCOVA showed no significant differences between the intervention and waitlist group completers when controlling for baseline anxiety symptoms, *F* (1, 48) = 0.581, *p* = 0.450, *n*_*p*_^2^ = 0.012. Adjusted means indicated that the intervention arm (*M* = 8.91, SE = 0.91) did not show a statistically significant difference in post‐intervention anxiety scores compared to the WLC (*M* = 10.62, SE = 0.73).

#### RCADS depression total

ANCOVA results did not produce a statistically significant effect, *F* (1, 48) = 0.939, *p* = 0.337, *n*_*p*_^2^ = 0.019. Examining adjusted means, the intervention group (*M* = 9.43, SE = 0.54), did not statistically differ on scores, compared to the WLC (*M* = 8.74, SE = 0.47).

#### AFQ total

A statistically non‐significant effect was observed, *F* (1, 44) = 0.036, *p* = 0.850, *n*_*p*_^2^ = 0.001, and when examining adjusted means the intervention group (*M* = 16.17, SE = 0.84) did not significantly differ compared to the WLC (*M* = 16.38, SE = 0.69).

#### IPTQ total

The ANCOVA showed no significant differences, *F* (1, 43) = 2.997, *p* = 0.091, *n*_*p*_^2^ = 0.065. The adjusted means indicated the intervention group (*M* = 8.6, SE = 0.78) did not significantly differ compared to the WLC (*M* = 10.43, SE = 0.61).

### Reliable changes

When inspecting the case completer data further, one (4%) of the participants in the treatment arm and one (3.4%) in the WLC demonstrated reliable improvement on the RCADS; two (9%) in the treatment arm and three (10.3%) in the WLC demonstrated reliable deterioration. No reliable improvement or deterioration was seen for AFQ scores in the intervention group, however one (3.6%) of the WLC reliably deteriorated. Furthermore, two (11.1%) experienced reliable improvement and one (5.6%) deteriorated in the intervention arm for IPTQ scores, whereas four (14.3%) improved and four (14.3%) deteriorated in the WLC.

## DISCUSSION

Building upon the feasibility trial by Perkins et al. (2021), this study aimed to investigate the efficacy of an online growth mindset SSI on reducing symptoms of anxiety and depression. In a sample of adolescents aged 14–18 years old, we found no evidence of treatment effect on anxiety and depressive symptoms, possibly due to a small sample size and a low retention rate. Nevertheless, this study represents one of the least expensive UK RCTs that is freely accessible for young people.

This study highlights the unique opportunities and challenges of recruiting young people to take part in online SSI RCTs. Whilst social media recruitment enabled us to reach adolescents *en masse*, this approach did not translate to full participation, even with the use of paid advertisement. This outcome highlights the need to consider other strategies to increase participant's engagement, such as behaviour change techniques (Michie et al., 2011), the use of regular reminders, and providing guaranteed incentives.

The intervention effects on anxiety and depressive symptoms were both statistically non‐significant. The null finding for anxiety at 1‐month follow‐up is consistent with the finding (−0.37, 95%CI [−0.81, 0.07]) reported by Perkins et al. (2021). A significant effect may be detectable in this study if anxiety‐related outcome was measured at a later follow‐up, as the study by Perkins et al. (2021) reported having a significant effect on anxiety symptoms at 8‐week follow‐up (−0.57, 95%CI [−1.02, −0.13]). This finding highlights the importance of measuring outcomes at multiple intervals over time.

The non‐significant effect on depressive symptoms is consistent with one study (Zimmermann & Papa, 2023), but differs from other studies (Osborn et al., 2020; Schleider & Weisz, 2016, 2018). This result suggests the effect of online SSI on depressive symptoms is not always replicable and may vary by other factors such as intervention design.

In terms of secondary outcomes, we found a significant effect favouring the intervention group for personality mindset, suggesting that the intervention did help participants to adopt a growth mindset. This finding is consistent with the outcomes reported by Perkins et al. (2021), who found a significant effect on personality mindset at 4‐ and 8‐week follow‐up. This is a promising finding, given prior research highlighting the positive links between a growth mindset and adolescent psychological wellbeing (Yeager & Dweck, 2023). To adjust for multiple comparisons, we applied Bonferroni correction, and the effect became statistically non‐significant. One possible interpretation is the intervention did what it was designed for, but its effect may be more detectable over a longer period.

In terms of reliable improvement in the case‐completer sample, we did observe some improvement across all outcome measures in the intervention arm (other than psychological flexibility, although we observed deterioration in the WLC). There were also improvements observed in the WLC, but a higher amount of deterioration in total. These findings, however, should be interpreted with caution given the study was underpowered.

### Strengths and limitations

The study had a small sample size and a high attrition rate, which are common in the context of online RCTs (Moffat et al., 2023; Team et al., 2018). The recruitment numbers are comparable to the numbers reported by Schleider and Weisz (2016), who randomised 96 young people and lost about half to follow‐up attrition over nine months. Furthermore, this study has built upon the feasibility trial of the growth mindset intervention which recruited 80 young people (retaining 52.5% at the 8‐week follow‐up).

In terms of attrition, it was effortless for participants to disengage with the RCT (e.g. closing a browser tab on their phone) which is both positive for freedom to choose to engage, but negative for retention and understanding why participants disengaged. Additionally, in the present study although we can be certain those in the intervention arm were on the intervention page for the required length of time, we cannot be certain the level of attention paid. It is also worth noting over half the young people recruited scored above cut off for anxiety and depression symptoms, compared to 20%–25% in the feasibility trial by Perkins et al. (2021). Neil et al. (2009) suggests that clinically higher scoring adolescents are more likely to complete an intervention in a naturalistic setting which was seen in the present study, with the high completion rate for those in the intervention arm. However, it is possible that the intervention appealed to young people who were experiencing a higher level of difficulties than this SSI is sufficient to meet, potentially accounting for poor retention to follow‐up. This also detracts from aims to test the SSI as a universal intervention to promote mental wellbeing. Another potential barrier to engaging with the follow‐up incentive was a low value prize draw. Cohen and Schleider (2022) found that RCTs were more likely to retain young people when they were paid to complete follow‐ups. A further limitation we did not account for was the delay in follow‐up completion. A participant who followed up on time may have experienced greater therapeutic effects than those who surpassed the 4‐week window.

The study's strength lies in its longitudinal design and participant's adherence to the intervention protocol, given that every randomised participant to the intervention arm took part. No young people contacted the research team to report adverse advents and there was also evidence of reliable improvement in the intervention arm, with only small evidence of reliable deterioration. The intervention was also previously deemed acceptable and feasible, and the engagement in the current trial is further evidence of this. Additionally, young people aged 14–18 years were able to access the intervention without parental consent and could access the research trial when it was suitable for them. We were also able to measure engagement in real world context. Another strength is that the trial adhered to its pre‐registration and conducted an ITT analysis. Small effects are not uncommon for brief universal interventions (Mackenzie & Williams, 2018) and slight changes could have wide‐reaching consequences over time at a population‐level (Funder & Ozer, 2019). The ITT analysis yielded a significant large effect for personality mindset, this does need to be interpreted with caution as the study was underpowered. However, missing data is a real issue for clinical trials and again not uncommon (Austin et al., 2021), and this study aimed to handle missing data transparently.

### Research implications

Future studies of SSIs need to improve recruitment rate and adherence to explore their true effects. One option is giving financial incentives, although this is less pragmatic as it would not be scalable in real world public health settings. Other options include recruiting targeted samples and making study advertisement more attractive to young people (e.g., co‐producing materials with young people to ensure they are appealing or using popular public figures to promote the study online). Explore other recruitment sites could be helpful, such schools, mental health services and youth activity groups (Hatch et al., 2023). Further research on clinical samples would be needed to find out whether such intervention are effective to them. Research may also identify moderators and mediators of growth mindset to see who is benefiting most from these interventions (Burnette et al., 2023).

### Clinical implications

Despite the modest results, our intervention demonstrates the potential scalability of digital guided self‐help approaches, which can be delivered to general population with ease, aligning with the growing recognition of digital mental health interventions for young people (NICE, 2023). Due to their flexible and accessible nature, brief digital SSIs could be potentially incorporated into community mental health services. These interventions could be particularly valuable for individuals on the treatment waiting lists (e.g., Fursland et al., 2018), relieving pressure on public health services.

### Conclusion

In summary, SSIs are emerging as a universal tool to promote mental health and a promising way to potentially meet the gap in mental health service provision. Given that most of the research in the field has been conducted in the United States where mental healthcare is not free and demand is high, it will be beneficial to continue exploring within a UK population to better understand how young people engage with it. The present research paves the way for future low‐cost RCTs to explore the efficacy of growth mindset SSIs for young people.

## AUTHOR CONTRIBUTIONS

**Jessica Ball**: Conceptualization; data curation; formal analysis; investigation; methodology; project administration; writing—original draft; writing—review and editing. **Richard Meiser‐Stedman**: Conceptualization; formal analysis; investigation; methodology; project administration; supervision; writing—review and editing. **Maria Loades**: Methodology; supervision; writing—review and editing. **Amorette Perkins**: Conceptualization; methodology; resources; supervision; writing—review and editing. **Gemma Bowers**: Conceptualization; resources; writing—review and editing. **Laura Pass**: Conceptualization; supervision; writing—review and editing. **Joseph Cassidy**: Resources; supervision. **Kenny Chiu**: Conceptualization; data curation; formal analysis; investigation; methodology; supervision; validation; writing—review and editing.

## CONFLICT OF INTEREST STATEMENT

The authors declare no conflicts of interest.

## ETHICAL CONSIDERATIONS

Ethical approval was granted by the University of East Anglia Faculty of Medicine and Health Sciences Research Ethics Committee (ETH2223‐0067).

## TRIAL REGISTRATION

The trial was registered with ClinicalTrials.gov (NCT05676554) before the first participant was recruited and followed CONSORT guidelines for reporting of RCTs.

## Supporting information

Supporting Information S1

## Data Availability

The data that support the findings of this study are available from the corresponding author upon reasonable request.
